# Sustainable Fully Printed UV Sensors on Cork Using Zinc Oxide/Ethylcellulose Inks

**DOI:** 10.3390/mi10090601

**Published:** 2019-09-12

**Authors:** Joana Figueira, Cristina Gaspar, José Tiago Carvalho, Joana Loureiro, Elvira Fortunato, Rodrigo Martins, Luís Pereira

**Affiliations:** i3N/CENIMAT, Department of Materials Science, NOVA School of Science and Technology, Universidade NOVA de Lisboa and CEMOP/UNINOVA, Campus de Caparica, 2829-516 Caparica, Portugal

**Keywords:** UV sensors, cork substrates, printing, low temperature, zinc oxide nanoparticles

## Abstract

Low-cost and large-scale production techniques for flexible electronics have evolved greatly in recent years, having great impact in applications such as wearable technology and the internet of things. In this work, we demonstrate fully screen-printed UV photodetectors, successfully fabricated at a low temperature on a cork substrate, using as the active layer a mixture of zinc oxide nanoparticles and ethylcellulose. The photoresponse under irradiation with a UV lamp with peak emission at 302 nm exhibited a quasi-quadratic behavior directly proportional to the applied voltage, with a photocurrent of about 5.5 and 20 μA when applying 1.5 V and 5 V, respectively. The dark current stayed below 150 nA, while the rise and falling times were, respectively, below 5 and 2 s for both applied voltages. The performance was stable over continuous operation and showed a degradation of only 9% after 100 bending cycles in a 45 mm radius test cylinder. These are promising results regarding the use of this type of sensor in wearable applications such as cork hats, bracelets, or bags.

## 1. Introduction

The smart wearable market is expected to surpass $120 billion over the next 5 years, while the internet of things (IoT) is expanding every year, with an unprecedented scale of trillions of connected devices in utilization globally possible as early as 2025 [1,2,3]. Alongside this growth, the search for materials and techniques that are simultaneously eco-friendly, sustainable, and low-cost to enable scaling up have become increasingly important.

Printing techniques are compatible with scalable and cost-effective processes, as well as being highly adaptable and much faster than conventional semiconductor processing technologies, which is a key factor for low-cost wearable electronics. When the use of low-environmental-impact materials (a consideration that is frequently neglected) is combined with low-energy processes (low processing temperatures, no vacuum systems), it results in the production of a more eco-sustainable device. Among several options available, screen-printing (SP) is one of the most commonly used printing methods due to the possibility of using it in a roll-to-roll process. Different type of sensors produced using this technique have been recently reported [4,5,6,7].

Cork is considered a sustainable material, currently used for different industrial applications, of which stoppers for the wine industry is one of the most representative. The cork forests constitute a unique and diversified ecosystem, and cork-based products are considered “carbon neutral” since they contribute to decrease the amount of carbon dioxide in the atmosphere, being long-life, biologically based products. Cork is harvested without killing the tree, allowing future bark extractions, and used cork can be easily granulated and recycled into raw material for a second life [8,9]. Macroscopically, cork is a lightweight material, with an average density of about 200 kg/m^3^, practically impermeable to liquids and gases, innocuous, and almost age-insensitive, with a remarkable chemical and biological stability along with a good resistance to fire. It is an elastic material, thermal and electrical insulator, and acoustic/vibration absorber. Microscopically, cork consists of layers of alveolar-like cells of which the membranes have a degree of waterproofing and are filled with an air-like gas, which occupies about 90% of their volume [10]. The number of published works focused on cork has increased over the years, which indicates a growing interest from the scientific community in this great raw material [8]. New applications of cork for sports, filters, pharmacology, fashion, and architecture, among others, have been developed and commercialized. From haute couture shoes to designer clothes, cork is being used as a raw material to enrich these goods, attracting customers who seek not only high quality products, but also goods from natural sources. The fabrics used to manufacture such commodities consist of extremely thin laminated sheets of natural or agglomerated cork bonded over cloth or another flexible support like paper [8]. Despite the emergence of these innovative applications, to our knowledge, there have been no reports on using cork sheet as a substrate for printed electronic. Printing sensors, antennas, and actuators, among other components, integrated into cork objects/accessories, would be a step forward for this “green” material to be used in the growing market for smart and wearable products.

In this work, we have demonstrated the feasibility of producing a UV sensor using a low-cost SP technique to deposit recyclable materials onto a flexible cork sheet substrate, without any previous surface treatment, using a low-temperature process. Zinc oxide (ZnO) is a well-known UV-radiation-photosensitive material, and is also a biocompatible and eco-friendly material [11,12,13]. Ethyl cellulose (EC), which is a widely available and recyclable cellulose derivative, was used as a thickening agent in the ZnO ink, to adjust its viscosity [14]. Commercial carbon ink was used for the electrodes printing, with advantages such as being less reactive and more resistant to oxidation than other inks.

## 2. Experimental Section

The ZnO ink vehicle was prepared by dissolving 5 wt. % ethylcellulose (C_6_H_7_O_2_(OC_2_H_5_)_3_; CAS:9004-57-3 from Aldrich, Alabama, AL, USA) in a 80:20 toluene/ethanol solution (both purchased from Aldrich, Alabama, AL, USA). After complete dissolution by stirring at 250 rpm overnight, ZnO nanopowder (<100 nm particle size, CAS:1314-13-2 from Aldrich, Alabama, AL, USA) was added, representing 40 wt. % of the final ink, and the mixture was continuously stirred until it was dispersed completely. ZnO/EC layers were screen-printed using a mesh model 77–55 (mesh count 190, aperture 81 μm, thread diameter 55 μm) and dried at room temperature. Carbon ink (TU-10s, 30–50 Pa.s) was obtained from Asahi Chemical Research Laboratory Co., Ltd (31350 Ipoh, Perak, Malaysia), and the pattern of the carbon interdigital electrodes was defined using a mesh model 120–34 (mesh count 305, aperture 45 μm, and thread diameter 34 μm). The pattern was dried at 100 °C. In this work, commercial cork sheet fabric obtained from Corticeira Viking Lda. was used as substrate.

For comparison purposes, reference samples were printed on a regular glass substrate 1 mm thick from Marienfeld (Lauda-Königshofen, Germany) (previously cleaned and then subjected to a UV-irradiation surface treatment lasting 30 min through a UV–ozone cleaner—Novascan PSD Series).

The photoresponsivity and time response of the prepared sensors were obtained using a Gamry Instruments Reference 600 Potentiostat in a chronoamperometry configuration, applying a continuous DC voltage (1.5, 5, or 10 V) between the interdigital electrodes. The response of the photodetector was tested under dark (UV lamp OFF) and light (UV lamp ON) conditions, using a UV lamp UVM-28; 8 W; λ = 302 nm (UVB) at a fixed distance of 10 cm from the sensor. The ON/OFF time period was 30 or 60 s, depending on the tests, and the time step for data acquisition was fixed at 0.1 s.

The surface of the substrate and printed layers was examined by scanning electron microscopy (SEM) using a Hitachi TM3030Plus table top workstation (Tokyo, Japan), to assess the printing quality and substrate coverage.

In order to study the sensors’ flexibility, two different mechanical tests were performed: a cycling test with a controlled bending radius (45 mm and 25 mm) and a fixed bending test (45 mm) over time (up to 48 h). A standard peeling test, also known as “Scotch Tape test” was performed using a commercial transparent adhesive tape from Staples® to make inferences about the adhesion of the printed layers to the cork. 

## 3. Results and Discussion

### 3.1. Substrate and Printing Process

**Figure 1:**
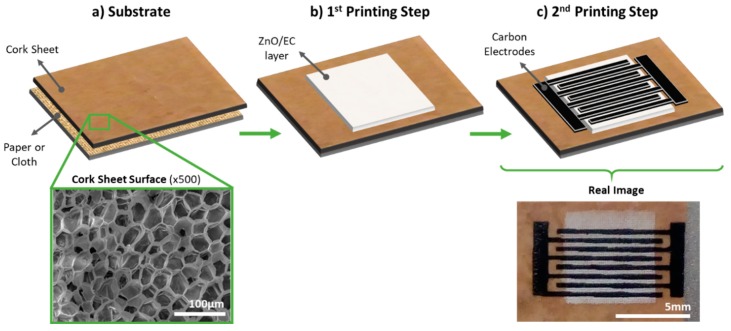
UV photosensor printing process scheme: (**a**) cork substrate glued to paper or fabric; (**b**) ZnO/EC (ethylcellulose) active layer printing step; (**c**) carbon interdigital electrode printing step. A scanning electron microscope (SEM) image of the cork surface is also shown, together with a top view of the sensors obtained by an optical microscope.

The UV photosensors were printed directly onto the cork sheet surface without any pre-treatment, as shown in Figure 1a. The ZnO/EC layers were the first to be printed, with a drying step at room temperature, followed by printing of the carbon interdigital electrodes, with a drying step of 30 min at 100 °C (Figure 1b,c, respectively). The photosensitive layer had the dimensions of a 6.5 × 6.5 mm square, and the electrodes had four pairs of interdigital fingers 0.3 mm wide, with a 0.3 mm gap between neighboring fingers, which left an active layer of approximately 13.65 mm^2^ to be directly stimulated by the light source. A real top-view image of a finished photosensor is shown in Figure 1c.

### 3.2. Electrical Characterization

#### 3.2.1. Number of printed ZnO/EC layers

**Figure 2:**
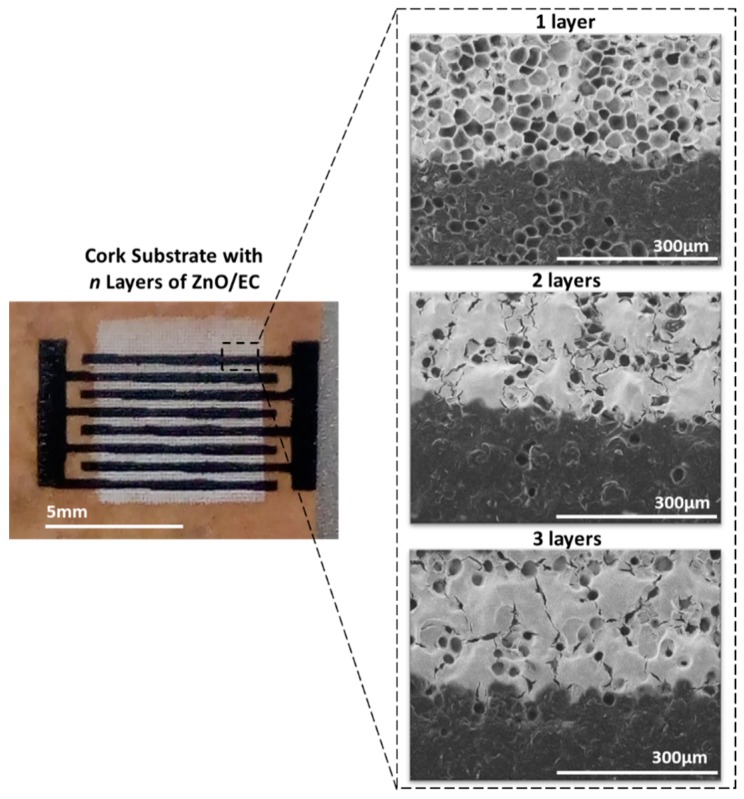
SEM images of the printed layers on top of cork sheet, showing the influence of the number of printed layers (n) on the coverage quality and surface morphology. The light zones correspond to the printed ZnO/EC, while the dark ones are the printed carbon interdigital electrodes.

**Figure 3:**
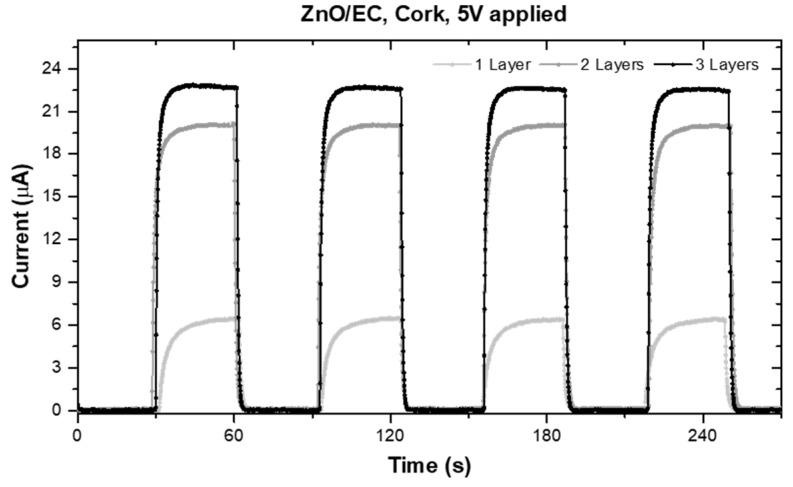
Photoresponse of printed UV-sensitive films, differing in the number of ZnO–EC layers.

The photosensors’ response to UV light was obtained for different numbers of printed ZnO/EC layers (one, two, and three layers, respectively). The total measurement time was the same for all the samples (four completed cycles), as well as the time steps for the ON/OFF periods (30 s). The voltage applied to the electrodes was fixed at 5 V for all the measurements. It was verified by SEM that one printed layer was not enough to cover the cork surface and smooth it (Figure 2), which was reflected in the sensor performance (Figure 3). It should be mentioned that the cork surface did not have any pre-treatment (a planarization/filling layer, for instance), which explains the difference observed in the morphology when increasing the number of printed layers.

Ideally, the sensor performance should translate into a quadratic photoresponse curve, evidencing fast response times to the ON/OFF UV lamp cycles. In our case, we had almost that ideal behavior, being more evident for the thicker samples (two and three layers). The photoresponse of the sensor with just one printed layer showed a less quadratic shape, with longer response times and a lower ON current (I_ph_) when compared with the others. This can be explained by the UV-sensing mechanism, which is based on the oxygen vacancies that exist on the surfaces of ZnO nanoparticles and that influence the electrical properties. As described in other works [12,13,15], without UV radiation, molecules containing high concentrations of oxygen are adsorbed at vacancy sites, capturing free electrons and depleting the particle’s surface, leading to an electrical conductivity reduction. On the other hand, under UV light exposure, with photon energy above the ZnO bandgap (3.3 eV), electron–hole pairs are photogenerated. These holes decrease the depletion zone by discharging the adsorbed negative ions, followed by desorption from the surface, which leaves behind free electrons, inducing an increase in the measured photocurrent. These phenomena of adsorption and desorption can be described by the following equations [13]:

(1) $$O_{2}\left(g\right)+{\;e}^{-}\to O_{2}^{-}\left(\mathrm{ad}\right)$$

(2) $$h\nu \to {\;e}^{-}+h^{+}$$

(3) $$h^{+}+O_{2}^{-}\left(\mathrm{ad}\right)\to {\;O}_{2}\left(g\right)$$

Since the above-mentioned phenomena are mainly surface related, the thicker layers did not show significant differences, from which we concluded that only two printed layers is enough for the production of low-cost UV photosensors, and will be faster and less wasteful. Thus, issues with solvent volatilization, which occurs between layers of printing, are reduced, avoiding possible microcrack formation [16].

#### 3.2.2. Voltage and Cycle Times

**Figure 4:**
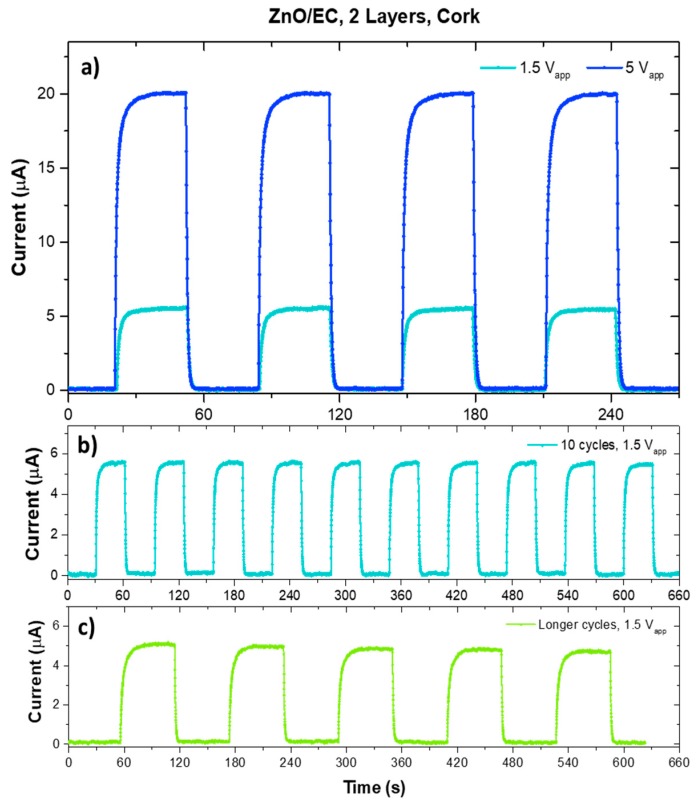
(**a**) Photoresponse of a cork sensor with two ZnO/EC printed layers for different applied voltages; (**b**) photoresponse of a cork sensor to 10 complete cycles ON/OFF, at a lower voltage; (**c**) photoresponse of a cork sensor to longer ON/OFF cycles.

Photoelectrical characterization was performed with varying applied voltages (1.5 or 5 V), with different ON/OFF time steps (30 or 60 s). As reported by Al-Hardan et al. [15], the response current was directly proportional to the applied voltage (Figure 4a), being I_ph_ ~5.5 μA when applying 1.5 V and I_ph_ ~20 μA when applying 5 V between the interdigitated electrodes.

The response time, which is reflected in the rise (τ_rise_) and fall (τ_fall_) times, was calculated by normalizing all the curves and measuring the time required for the I_ph_ to increase from 10 to 90%, and then to decrease from 90 to 10% of its maximum value. As can be seen in Table 1, both τ_rise_ and τ_fall_ decrease when the voltage applied between the electrodes is higher, as it was expected [15].

The performance was stable over time in continuous operation of 10 consecutive cycles of 30 s ON and 30 s OFF, with a lower applied voltage of 1.5 V, as seen in Figure 4b. In order to evaluate the photosensor behavior during longer cycles, we measured the response using ON/OFF cycles of 60 s instead of 30 s (Figure 4c). All the measured printed sensors responded well to UV light irradiation, showing fast response times and low dark currents (I_dark_ < 150 nA), as well as a homogeneous behavior over cyclic stimulation time. These features were comparable or even better than those of similar photodetectors reported in the literature, including some produced with highly complex and expensive techniques using vacuum systems and high temperatures, processes that are not compatible with flexible and lightweight substrates and thus wearable technology [5,11,15,17,18,19,20,21,22].

#### 3.2.3. Cork vs. Glass Substrate

**Figure 5:**
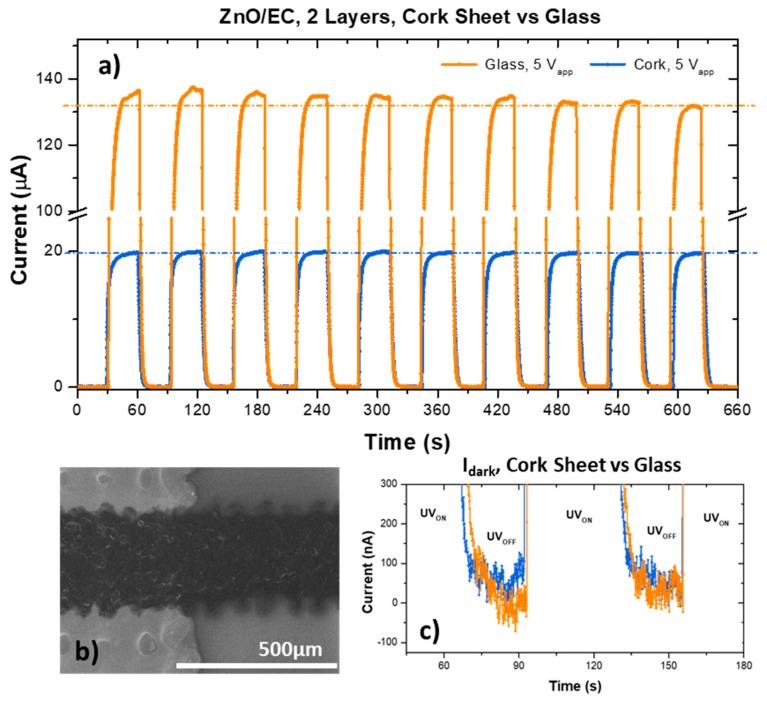
(**a**) Photoresponse of two sensors, one printed on a glass substrate and the other on a cork substrate; (**b**) SEM image of the printed sensor surface using the glass substrate; (**c**) zoomed in view of the dark currents.

The printing process was replicated on a non-porous and rigid glass substrate (1 mm thick, cleaned and UV light pre-treated for 30 minutes), used as a reference. The electrical response results are shown in Figure 5, compared to the ones obtained for printed sensors on cork. The maximum photocurrent (~130 μA) obtained in the sensor printed on glass was nearly seven times higher than that of the ones printed on cork (Figure 5a). This difference was attributed to the quality of both the ZnO active layer and the printed electrodes on the different substrates (Figure 5b vs. Figure 2). Since the cork sheet surface did not undergo any planarization pre-treatment, the glass was expected to have much smoother, homogeneous and continuous layers, allowing a better performance. Nevertheless, the response times and dark current (Figure 5c) values were similar, suggesting that a better layer formation also allowed for surface sites to actively respond to UV irradiation, with stronger influence on the I_ph_.

#### 3.2.4. Adhesion, Bending, and Durability

**Figure 6:**
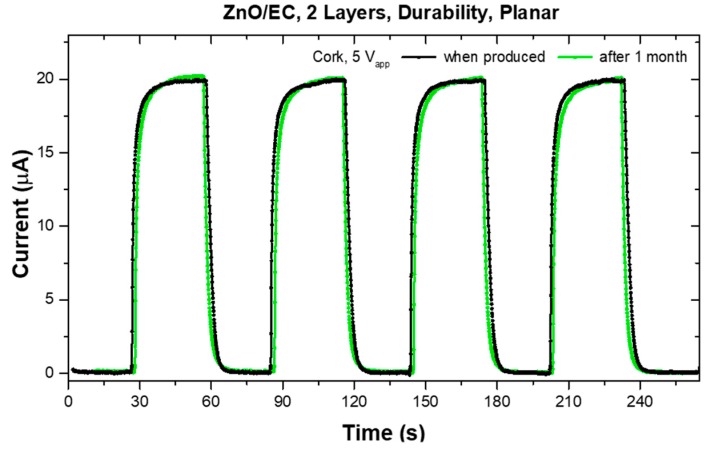
Photoresponse of a cork sensor over time, right after being produced and then after one month of being stored in the lab (23 °C and 30% RH).

Different mechanical tests were performed in order to evaluate the suitability and endurance of the UV photosensors printed on cork sheet substrates while in use in wearable applications. The samples were submitted to adhesion and bending tests right after being produced, and then again after one month of being stored in the lab (23 °C and 30% RH), to also draw inferences about durability over time. As shown in Figure 6, we confirmed the environmental stability of the printed sensors on cork, since the response under UV light was maintained.

**Figure 7:**
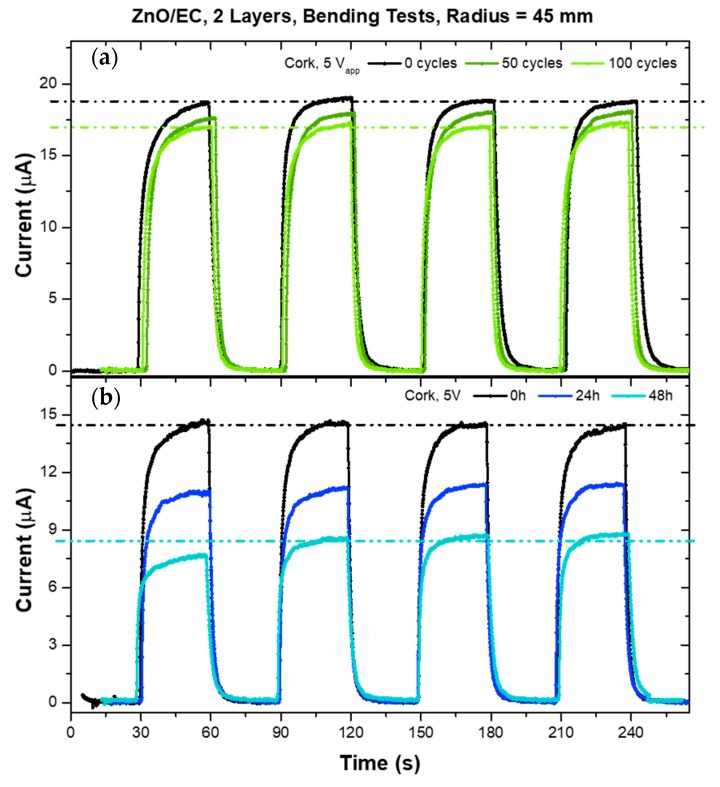
(**a**) Photoresponses during cyclic bending deformation using a cylinder with a 45 mm radius; (**b**) Photoresponse for a fixed curvature over time on the same test cylinder.

Figure 7a shows the results for a cyclic bending deformation using a cylinder with a 45 mm radius. The black curve shows the photosensitivity response of the printed sensor before any mechanical stress, while in planar form. After being submitted to 50 bending cycles, the sensor was characterized again in its planar form (dark green curve), showing a decrease of ~5% in the photocurrent. Adding 50 more bending cycles, a decrease of ~9% relative to the initial photocurrent was observed, showing a slight degradation attenuation (light green curve). After these 100 bending cycles, the same sensor was fixed to the 45 mm radius cylinder and measured over time in that curved form (Figure 7b). Although the sensor was degraded when curved over time, it still presented ~50% I_ph_ response to the UV light after 48 h when compared to the initial state before bending. When the bending curvature radius was reduced to 25 mm, the mechanical stress appeared to be much more harmful to the UV sensors, with a decrease in I_ph_, of ~25% and ~43% after 50 and 100 bending cycles, respectively.

A peeling test was performed to check the adhesion of the printed layers onto the substrate (and between layers), with the cork sensor photosensivity measured before and after the test. Before and after the peel, the photocurrent suffered a variation of just ~2%, and the microscope images revealed no significant modifications of the sensor surface, confirming a good adhesion of the printed layers onto the substrate. (Results not shown.)

## 4. Conclusions

In this work, we have reported for the first time the fabrication of fully-printed UV photosensors on an eco-friendly and low-cost cork sheet substrate. Proper optimization of the ZnO/EC ink and number of printing steps allowed any pre-treatment of the substrate surface to be avoided, while the whole production process was done at a maximum temperature of 100 °C. The EC was revealed to be a good option as a binder and dispersing agent for ZnO nanoparticles. The combination of the eco-friendly functional ink with a top-electrode architecture allowed for a fast response to UV irradiation with a τ_rise_ < 5 s and τ_fall_ < 2 s. The photo-response of the sensors on cork remained stable for more than one month without any encapsulation or special storage conditions. The results presented here show that cork is an alternative low-cost and sustainable substrate able to be used in printed electronics, presenting a great potential for wearable applications. 

## Figures and Tables

**Table 1:** UV sensor response times for 1.5 V and 5 V applied between the electrodes.

Applied Voltage (V)	Rise Time (s)	Fall Time (s)
1.5	4.4 ± 0.5	1.9 ± 0.1
5.0	3.6 ± 0.2	1.5 ± 0.1
